# Reply to the Letter to the Editor: Diagnostic performance of the clear cell likelihood score integrated with cystic degeneration or necrosis on MR imaging for identifying clear cell renal cell carcinoma in cT1 solid renal masses

**DOI:** 10.1186/s13244-026-02315-3

**Published:** 2026-05-29

**Authors:** Xueyi Ning, Haiyi Wang

**Affiliations:** 1 Department of Radiology, The First Medical Center of the Chinese PLA General Hospital, 28 Fuxing Road, Haidian District, Beijing, China; 2 Chinese PLA Medical School, 28 Fuxing Road, Haidian District, Beijing, China

We thank Dr. Hekimsoy for his interest in our recent publication, “Diagnostic performance of the clear cell likelihood score integrated with cystic degeneration or necrosis on MR imaging for identifying clear cell renal cell carcinoma in cT1 solid renal masses” [1]. We appreciate the opportunity to address the points raised in his letter.

First, Dr. Hekimsoy raises an important pathological perspective regarding cystic degeneration and necrosis. We agree that these two findings are not equivalent at the pathological level, and we did not intend to regard them as biologically identical. In this study, cystic degeneration and necrosis were used as MRI assessment indicators based on previous literature and clinical experience [1, 2]. As described in our article, both findings manifest as non-enhancing areas within the tumor on MRI, and there is overlap between them on cross-sectional imaging, making strict differentiation difficult in clinical practice, especially for less experienced readers [1]. Therefore, we combined cystic degeneration and necrosis as an imaging feature in the ccLS integrated with cystic degeneration or necrosis (cn-ccLS), rather than as a pathological classification. This design was also supported by the interobserver agreement results in our study. The *κ*-values of cystic degeneration, necrosis, and cystic degeneration or necrosis were 0.64, 0.49, and 0.76, respectively, and the overall *κ*-value improved from 0.62 for ccLS to 0.71 for cn-ccLS [1]. This may suggest that combining the two findings improves the reproducibility of MRI assessment. At the same time, we agree that the imaging–pathology correlation of cystic degeneration and necrosis still requires further study, as noted in the Discussion and Limitations sections of our article.

Second, regarding diagnostic spectrum bias, we acknowledge this concern and agree it is clinically relevant. Our study included only the five common renal tumor subtypes targeted by the clear cell likelihood score (ccLS) system and excluded other rare or emerging renal tumor types, including those mentioned in the letter [1, 3, 4]. This is because the ccLS system itself was primarily developed for the most common renal tumor subtypes in its intended application scenario, and its diagnostic performance for rare subtypes has not yet been fully validated [3]. Since the cn-ccLS was developed based on the ccLS system, we followed the same framework in order to maintain methodological consistency and reproducibility. We agree that this design limits the generalizability of our findings to a broader pathological spectrum. However, we believe that this issue is better interpreted as a limitation of external validity, rather than direct evidence that the specificity of cn-ccLS was overestimated. The diagnostic performance of cn-ccLS in more heterogeneous populations still requires further validation in future multicenter studies, including a wider range of histological subtypes.

Third, we agree that, for many cT1b tumors, urologists generally tend to decide on surgical intervention according to the overall oncologic risk, and preoperative subtype characterization may not always change the final treatment strategy [5, 6]. However, we believe that the improved sensitivity of cn-ccLS in cT1b masses remains clinically significant. In our opinion, the value of preoperative identification of clear cell renal cell carcinoma (ccRCC) extends beyond whether surgery is performed and may also be reflected in improved diagnostic confidence, thereby assisting multidisciplinary discussion, supporting patient counseling, and providing additional information for preoperative risk assessment. In this study, we performed subgroup analyses of cT1a and cT1b masses primarily to evaluate the diagnostic performance of cn-ccLS across different T stages, rather than to suggest that surgical decisions should be changed solely on the basis of cn-ccLS. Therefore, we agree that the direct impact of cn-ccLS on treatment decision-making still needs to be confirmed by future prospective studies.

Finally, we agree with Dr. Hekimsoy that positive predictive value and negative predictive value are influenced by disease prevalence and should be interpreted in the context of the study population. In our study, the proportion of ccRCC was relatively high (78%), which may have contributed to the high positive predictive value and relatively low negative predictive value [1]. We agree that predictive values may differ when cn-ccLS is applied in other clinical settings with different prevalences. However, our study focused on pathologically confirmed cT1 solid renal masses without macroscopic fat within the ccLS application framework, rather than all renal masses encountered in routine clinical practice. Therefore, caution is needed when directly recalculating predictive values using prevalence estimates derived from populations that do not fully match our inclusion criteria. In our view, sensitivity and specificity may be more suitable than predictive values for comparison across different cohorts, although these parameters also need further external validation.

In summary, this study suggests that cn-ccLS is an MRI-based extension of ccLS that improves the sensitivity for identifying ccRCC in cT1 solid renal masses while maintaining similar specificity in our cohort. At the same time, we agree that its interpretation and clinical application should remain within the study population and framework in which it was developed. Future studies with larger samples, multicenter data, and a broader range of pathological types are still needed to further validate the generalizability and clinical value of cn-ccLS.

## Data Availability

Not applicable.

## Declarations

### Methodology

- Letter to the Editor
